# Antitumoural Sulphur and Selenium Heteroaryl Compounds: Thermal Characterization and Stability Evaluation

**DOI:** 10.3390/molecules22081314

**Published:** 2017-08-08

**Authors:** Verónica Alcolea, Pablo Garnica, Juan A. Palop, Carmen Sanmartín, Elena González-Peñas, Adrián Durán, Elena Lizarraga

**Affiliations:** 1 Department of Organic and Pharmaceutical Chemistry, Faculty of Pharmacy and Nutrition, University of Navarra, Irunlarrea 1, 31008 Pamplona, Spain; valcolea@alumni.unav.es (V.A.); pgarnica@alumni.unav.es (P.G.); jpalop@unav.es (J.A.P.); mgpenas@unav.es (E.G.-P.); elizarraga@unav.es (E.L.); 2 Department of Chemistry, Faculty of Sciences, University of Navarra, Irunlarrea 1, 31008 Pamplona, Spain; adrianduran@unav.es

**Keywords:** thermal analysis, activity, stability, polymorphism, differential scanning calorimetry, thermogravimetry, X-ray diffraction, HPLC

## Abstract

The physicochemical properties of a compound play a crucial role in the cancer development process. In this context, polymorphism can become an important obstacle for the pharmaceutical industry because it frequently leads to the loss of therapeutic effectiveness of some drugs. Stability under manufacturing conditions is also critical to ensure no undesired degradations or transformations occur. In this study, the thermal behaviour of 40 derivatives of a series of sulphur and selenium heteroaryl compounds with potential antitumoural activity were studied. In addition, the most promising cytotoxic derivatives were analysed by a combination of differential scanning calorimetry, X-ray diffraction and thermogravimetric techniques in order to investigate their polymorphism and thermal stability. Moreover, stability under acid, alkaline and oxidative media was tested. Degradation under stress conditions as well as the presence of polymorphism was found for the compounds VA6E and VA7J, which might present a hurdle to carrying on with formulation. On the contrary, these obstacles were not found for derivative VA4J.

## 1. Introduction

Cancer is a leading cause of death worldwide; the World Health Organization estimates the growth rate of new cases to peak at 70% in the next twenty years [1]. If we draw the focus to US territory, an estimated 1650 deaths per day will be due to cancer [2]. Many treatment patterns include some kind of chemotherapy or molecularly-targeted therapy drugs. Cancer research is nowadays encountering two major obstacles, side-effects and tumour resistance, both intrinsic and acquired [3,4]. Therefore, the synthesis of new, safer and more effective drugs is urgent in order to be able to overcome those previously mentioned difficulties developed by some of the present treatments.

Among the arsenal of new anti-cancer agents, selenium (Se)-containing compounds stand out for their anti-tumour potency and safety [5]. These properties strongly depend on the chemical form and dose used [6]. For the past decade, our group has focused on the synthesis of sulphur (S) and Se-containing molecules as antitumour agents with encouraging results [7,8]. In the present study, the homology between those two atoms will be explored in order to widen the variety of chemical entities. The introduction of this structural duality will enable us to contrast Se and S influence in terms of anti-cancer activity, stability and thermal characterization.

It has been found that pharmaceutical compounds such as cephalexin, chloramphenicol, digoxin, spironolactone, sulfathiazole, carbamazepine and retroviral agents [9,10,11,12] undergo transitions during drying processes, spraying, grinding, compression, excipient addition and the preparation of tableting. Polymorphism in pharmaceutical solids can be a critical manufacturing variable and therefore has to be investigated [13].

Physicochemical properties such as bioavailability and stability are directly related to the crystal structure and possible polymorphism [14,15]. Therefore, the prediction of polymorphism in the early stages of drug development provides valuable information to selectively synthesize or isolate the crystalline structure with the best characteristics. In addition, polymorphic transformations can occur during some steps of the synthesis including purification, crystallization, drying and storage [16,17]. Furthermore, this property influences several parameters such as solubility, shelf life, and formulation processes [18].

Investigating the relative thermal stability of polymorphs is also essential because the instant appearance or disappearance of a crystalline structure can lead to serious consequences if the polymorphic transformation occurs in the dosage form [19]. Da Silva et al. [20] have found that the solubility and dissolution properties of finasteride, which is widely used for prostate cancer treatment, were significantly affected by polymorphism. This disparity in bioavailability is related to the diverse dissolution rate of the different polymorphic forms. In some cases, the metastable form of some active ingredients may be twice as active as the stable form [21]. In addition, Yoshida et al. [22] have observed that the degradation products after drug stress conditions are related to some events that appeared in the calorimetric curves.

The study of the physical stability of drugs should be properly conducted in order to determine the characteristics of the proposed formulation and predict possible changes in the solid state of the drug. Certain pharmaceutical processes (granulation, drying, and compression) can lead to polymorphic transformations and thus the critical steps in this process must be identified and controlled to ensure stability until the end of the drug’s shelf life [23].

If we narrow the view to Se and S derivatives, those properties have proved to be critical. Some Se-containing structures i.e., ebselen, produce two different polymorphs when crystallized from different solvents that differ in the antioxidant activity related to the ability of Se–N bond cleavage as well as the potential for Se–O interactions [24]. Moreover, S derivative compounds such as dalcetrapib [25], probenecid [26] or tauroursodeoxycholic acid [27] showed the ability to crystalize in different structures, an ability that dramatically affects their biological activity profile.

Those heteroaryl derivatives that exhibited promising cytotoxic activity (VA7J, VA6O and VA5P) in previous publications from the group [28,29] have the potential to continue the drug discovery process. With the objective of predicting the non-desired transitions or degradation processes that may occur along with the already mentioned pharmaceutical manufacturing processing, we proceeded to the thermal characterization and preliminary stability studies of the leader compounds. In addition, the biological and thermal characterization of the novel compounds recently developed by our group have also been included in this work. Inactive analogues were also studied following the same methodology in order to be able to establish a comparison.

## 2. Results and Discussion

### 2.1. Synthesis and Selection Criteria

**Figure 1:**
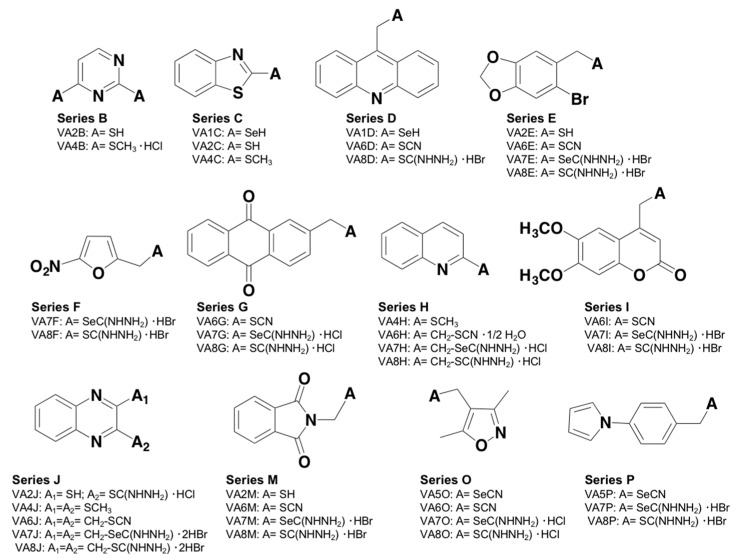
Structures of S and Se heteroaryl compounds studied in this work. The compounds VA6G, VA6H, VA6I, VA2J, VA4J and VA6J are novel molecules. The structures of series 7 and 8 have been previously described [29] and the remaining ones have been extracted from a previous work from our group [28].

The selected compounds for this study were synthesized in the Organic and Pharmaceutical Synthesis Unit of the University of Navarra. A total of 40 compounds were included in this work. They were divided into 12 series (series B to P, Figure 1) according to their central skeleton. They are hybrid derivatives containing diverse substituents with S or Se and several active heterocyclic scaffolds. Six novel derivatives (VA6G, VA6H, VA6I, VA2J, VA4J and VA6J) were incorporated to complete the series. The compounds VA6G, VA6H, VA6I and VA6J were synthesized according to a previously published procedure by reaction of the corresponding alkyl halides with potassium thiocyanate in acetone under reflux conditions [30]. To obtain compound VA2J, 2,3-dichloroquinoxaline was treated with thiourea in a 1:2.2 (reagent/thiourea) molar ratio at reflux, using absolute ethanol as solvent. VA4J was synthesized by the treatment of derivative VA2J with a potassium hydroxide solution in DMSO and stirring at room temperature (r.t.), with a posterior methylation with methyl iodide. The final compound was purified taking advantage of its solubility properties. The synthesis of the remaining 34 derivatives has been previously published by our group [28,29].

In order to perform the thermal characterization and preliminary stability studies of those compounds with the best drug-likeness profile, the cytotoxic potency was evaluated by the MTT (3-(4,5-dimethylthiazol-2-yl)-2,5-diphenyltetrazolium bromide) assay.

The novel synthetic compounds VA2J, VA4J, VA6G, VA6H, VA6I and VA6J were tested in breast adenocarcinoma (MCF-7) and non-malignant mammary-gland-derived (184B5) cell lines to be able to establish their potency and selectivity. The reference drug used was methylseleninic acid (MSA) as a Se-containing structure with promising in vitro and in vivo results against different tumour types [31,32]. Results are expressed as the concentration that inhibits 50% cell growth (GI_50_), the concentration that inhibits completely cell growth (TGI) and the concentration that kills 50% of cells (LD_50_). The selectivity index (SI) is calculated as GI_50_(184B5)/GI_50_(MCF-7).

As shown in Table 1, five out of the six tested compounds (VA2J, VA4J, VA6G, VA6H and VA6I) exhibited GI_50_ values under 10 μM. If we move to selectivity values, the compound VA4J emerges as the most selective derivative with an SI over 50 times higher than MSA.

For the remaining compounds of series **1**, **2**, **3**, **4**, **5** and **6**, cytotoxic activity had already been evaluated following the same methodology [28]. Cell viability assays for compounds of series **7** and **8** had already been screened in a panel of six human cancer cell lines: 1205Lu (melanoma), A549 (lung), DU145 (prostate), HCT116 (colon), PANC-1 (pancreas) and BxPC3 (pancreas) [29].

Based on all the synthetic data and antitumour preliminary activity evaluation, the following criteria were established to select the most promising structures to proceed to their thermal characterization and preliminary stability studies:

- Synthetic yield has to be higher than 40% as lower yields might present limitations to a future production scale-up.
- High activity against cancer cells must be exhibited.

Taking into account these criteria, the structure VA4J was chosen as the most promising one of the newly studied structures with a yield of 53%, GI_50_ value against MCF-7 cells under 10 μM and an SI of 51.25.

Derivative VA7J was selected from series **7** and **8** with 82% yield and the most potent activity profile. Even though the MTT assay conditions were slightly different in this work, a clear leader can be established. Compound VA7J was selected from this group of structures as it shows IC_50_ values under 10 μM in all six cell lines even at short time points.

Focusing on the compounds remaining of series **1**, **2**, **3**, **4**, **5** and **6**, compounds VA6O and VA5P were selected in the referenced work as the most potent ones. Nevertheless, VA6O and VA5P had to be dismissed for this study as VA6O has an oily physical appearance and VA5P showed fusion with decomposition in a preliminary test. Therefore, we followed the criteria of SI, potency and potential safety, to select the representative compound for these structures. According to these criteria, derivatives VA2E and VA6E were chosen. Compound VA2E did not meet the yield condition with a 5% yield, so it was ruled out. Finally, the compound VA6E was selected as it has a reasonable yield, high potency and it is fourteen times more selective than MSA [28].

### 2.2. Compound Thermal Characterization

The combination of several techniques is necessary in order to properly confirm polymorphism presence and thermal stability. The methodology employed to perform the complete thermal characterization of a compound include IR spectroscopy, powder X-ray diffractometry (PXRD) and thermal methods, especially differential scanning calorimetry (DSC) and thermogravimetry (TG).

#### 2.2.1. Compound VA6E

**Figure 2:**
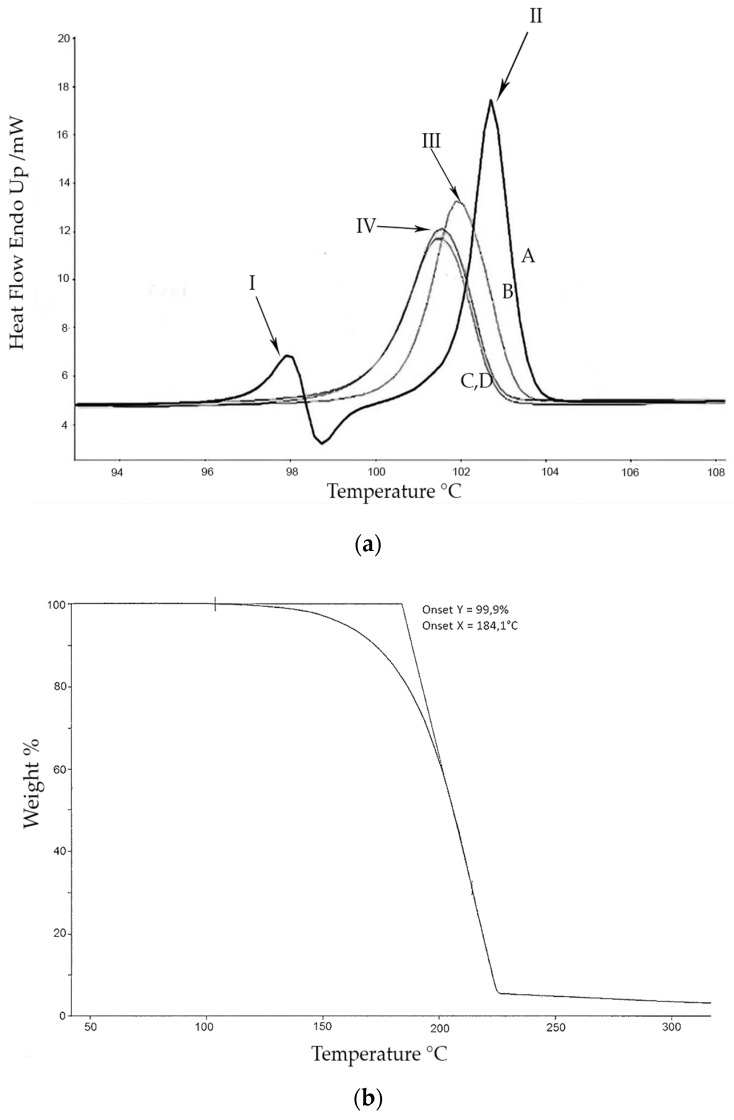
DSC and TG of compound VA6E (**a**,**b**) respectively. (**a**) Curve A: first scan (fusion Form I and Form II); curve B: second scan (fusion of Form III) and curves C and D: third and fourth scan (fusion of Form IV); (**b**) TG of compound VA6E at 10 °C/min.

The DSC results of compound VA6E are shown in Figure 2a and Table 2. During the first thermal scan (curve A), three processes were observed. The first corresponds with a typical endothermic process of the original product (Form I), followed by a recrystallization process to another form (Form II), which then melts. After cooling, in the sample—through a polymorphic phase transition—Form II changes into another form which shows fusion processes (Form III, curve B). In the third scan a new endothermic process happened at a lower temperature (curve C), with the appearance of a new polymorph (Form IV). The complete thermogram (Figure 2b) shows that other processes at another temperature (higher or lower) can be excluded.

**Figure 3:**
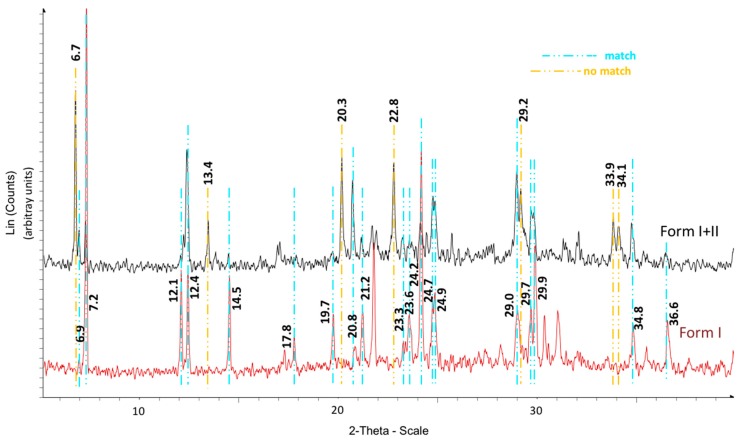
X-ray diffractograms for compound VA6E. Form I shows the diffractogram of the original polymorph and Form I + II, the one obtained after one cycle of melting-recrystallization.

Marked differences were found in the positions of the peaks between the diffractograms of the different forms. In Figure 3, the diffractogram in black corresponds to the sample after melting and the subsequent recrystallization. Some of the peaks matched with those from the original sample (diffractogram in red). However, other peaks only appeared after melting and recrystallization (2θ = 6.7°, 13.4°, 20.3°, 22.8°, 29.2°, 33.9° and 34.1°). In this case, we think Form I has partially transformed into another polymorphic form (Form II), while preserving part of the original form.

**Figure 4:**
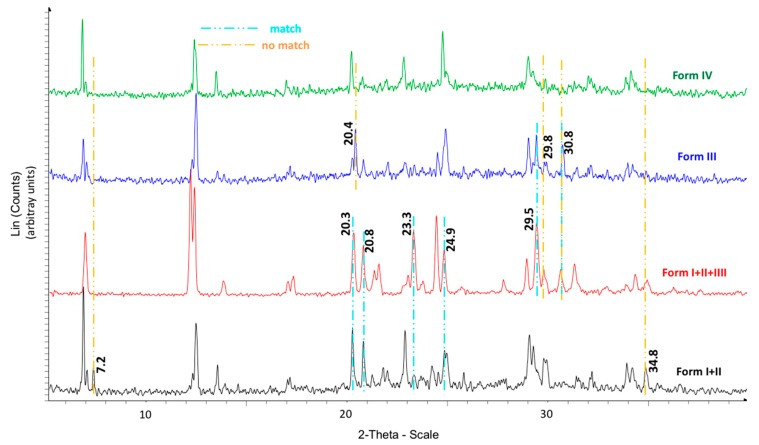
X-ray diffractograms of the forms of VA6E obtained after successive cycles of melting–recrystallization.

Figure 4 shows the X-ray diffractograms after different and consecutive melting-recrystallization cycles. As discussed before, after one cycle, a mixture of Forms I and II can be observed. The following cycle provoked the appearance of a new polymorphic form (Form III), but the original Forms I and II are also present (diffractogram in red) as the match of the position of the peaks 2θ = 20.3°, 20.8°, 23.3°, and 24.9° shows. Form III (diffractogram in blue) showed a peak at 2θ = 20.4° that was not detected in Forms I and II, as well as the absence of other signals present in Forms I and II (peaks at 2θ = 7.2° and 34.8°). Finally, Form IV was observed after another cycle (diffractogram in green). The differences between the resulting form from this last cycle and the previous ones are highlighted in yellow dotted lines. Peaks at 2θ = 20.4°, 29.8° and 30.8° are present in Form III but not in the last polymorphic form. Peaks at 7.2° and 34.8°, present in Form I + II, do not appear in Form IV. After successive heating–cooling processes the same Form IV was isolated. Taken together, the results of the X-ray diffraction indicate that all four forms observed by DSC were indeed distinct.

#### 2.2.2. Compound VA4J

**Figure 5:**
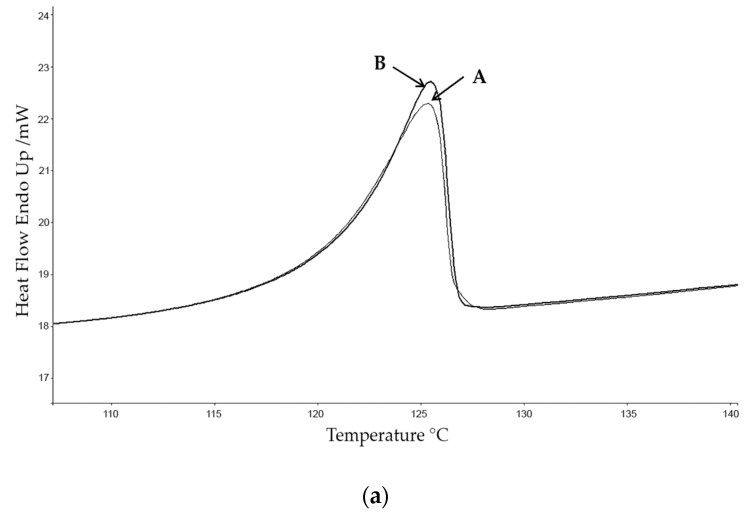

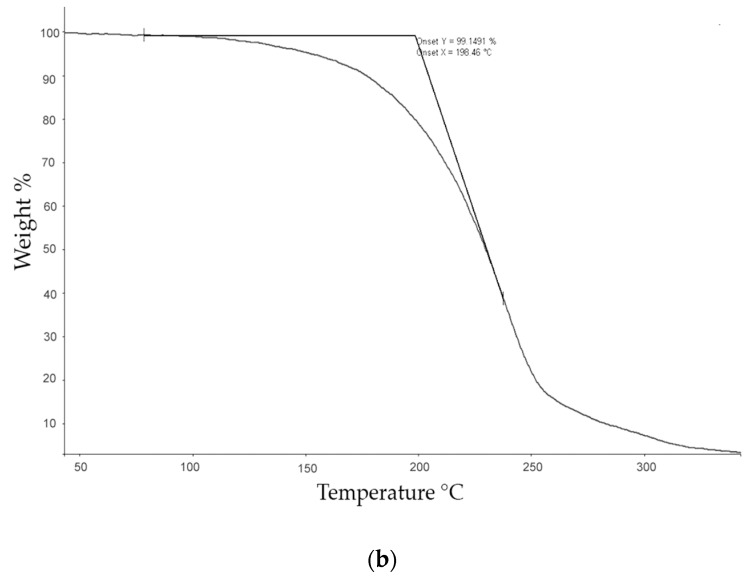
DSC (**a**) and TG (**b**) of compound VA4J. (**a**) Curve A: first scan (fusion of Form I) and curve B: second scan (fusion of recrystallized form); (**b**) TG of compound VA4J at 10 °C/min.

DSC results (Figure 5a and Table 3) show that the compound VA4J does not alter its thermal behaviour after an initial fusion–recrystallization cycle. Under these conditions there is no evidence of polymorphism. During the first thermal curve of compound VA4J (curve A in Figure 5a), a classic endothermic process of Form I was discerned (Table 3), typical of a melting process. After cooling the sample, the melted form recrystallized in the same form, as indicated by the same endothermic process in curve B.

**Figure 6:**
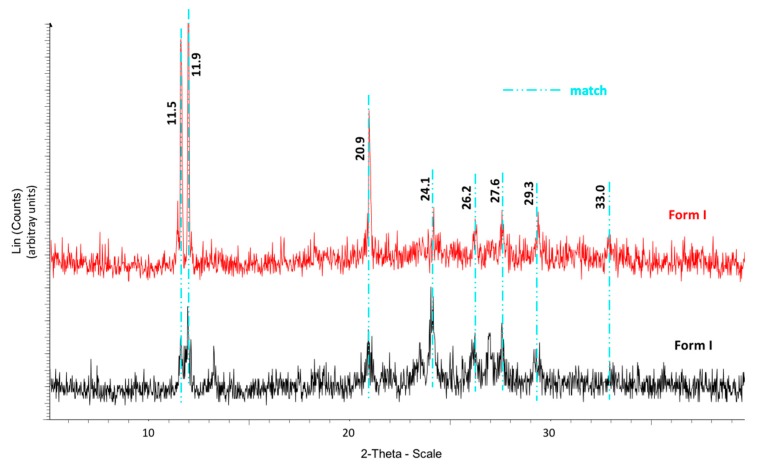
X-ray diffractograms of the original form of VA4J (in black) and those obtained after successive cycles of melting–recrystallization (in red).

To further confirm the absence of polymorphism, both samples were analysed by using PXRD. An excellent match of the position of the peaks in both diffractograms was observed (see peaks at 2θ at 11.5°, 11.9°, 20.9°, 24.1°, 26.2°, 27.6°, 29.3° and 33.0° in Figure 6). The differences in the relative intensities of some of the peaks were probably due to some preferred orientation in the samples derived from the absence of random orientation of crystal grains in space.

#### 2.2.3. Compound VA7J

**Figure 7:**
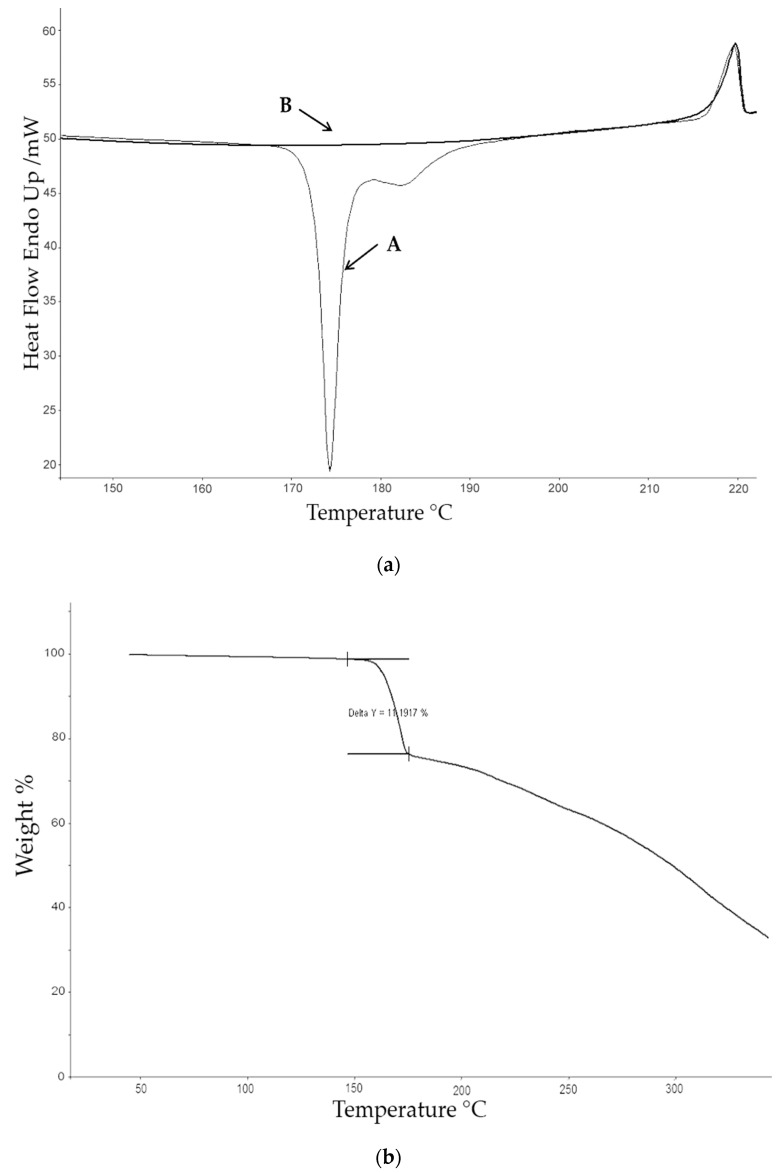
DSC (**a**) and TG (**b**) of compound VA7J. (**a**) Curve A: first scan (desolvation) and curve B: second scan; (**b**) TG of compound VA7J at 10 °C/min.

In the first heating scan, the original form of compound VA7J melts with desolvation. A new endothermic fusion of the anhydrous product generated confirms the polymorphism (pseudopolymorphism or phase transition of the hydrated form to the corresponding anhydride) of this compound (curve A, Figure 7a). After cooling the sample, it can be observed (curve B) that the subsequent endothermic process occurs at the same temperature (curve B, Figure 7a, Table 4).

**Figure 8:**
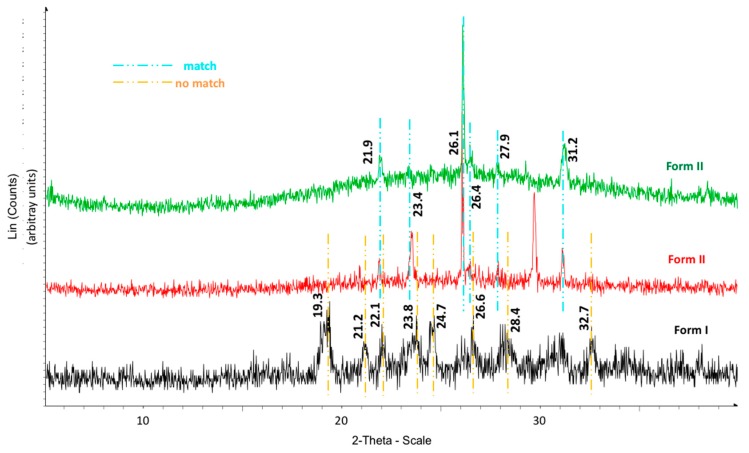
X-ray diffractograms of the forms of VA7J obtained after successive cycles of melting-recrystallization.

Two different compounds were detected in the sample VA7J by using PXRD (Figure 8). Several differences were found between the peak positions in the raw sample (diffractogram in black) and the sample after one desolvation/phase transition process (diffractogram in red). Peaks at 2θ = 19.3°, 21.2°, 22.1°, 23.8°, 24.7°, 26.6°, 28.4° and 32.7° were present in the original compound (Form I) but disappeared in Form II of anhydre compound. This confirms the appearance of a new polymorphic form.

The diffractogram collected after a melting scan and a subsequent recrystallization process of Form II of the compound (diffractogram in green) is very similar to the one obtained from the first cycle (in red). Therefore, a new polymorphic form is not present. In Figure 8, the majority of the diffraction peaks match in the 2θ position (see peaks at 2θ = 21.9°, 23.4°, 26.1°, 26.4°, 27.9° and 31.2°) and the observed differences between both diffractograms are in terms of the relative intensities.

### 2.3. Compound Stablity Tests

#### 2.3.1. Thermal Stability

Thermogravimetric analyses were performed to evaluate the thermal stability of the compounds (Figure 2b, Figure 5b and Figure 7b, Table 5. The TG analysis of the compounds VA4J and VA6E show that the decomposition or desolvation processes in the temperature range studied (100–135 °C and 94–108 °C, respectively) can be excluded. On the other hand, the thermogravimetric curve of the compound VA7J shows a debromhidration process at 173 °C, and when further heated, decomposition of desolvated compound from 228 °C.

#### 2.3.2. Stability after Stress Conditions

The stability of compounds VA4J (without polymorphic behaviour) and VA6E and VA7J (with polymorphic behaviour) was investigated after the application of diverse stress conditions: acid hydrolysis (HCl 1 M), basic hydrolysis (NaOH 0.1 M) and oxidation (H_2_O_2_ 3%). Chromatographic studies using HPLC/UV-DAD were performed with this purpose.

During calibration sample analysis, compound VA4J had a retention time of 4.9 min, compound VA6E of 7.3 min (for both Form I and IV), and compound VA7J of 1.1 min (for both form I and II).

The linearity showed an adequate relationship between the instrumental response (peak areas) and the respective sample concentration (*x*). Intra-day precision (Relative Standard Deviation (RSD) < 7%) was satisfactorily obtained in the ranges evaluated for the three compounds (Table 6). When new peaks appeared after stress conditions, they were adequately separated from the parent peak.

**Figure 9:**
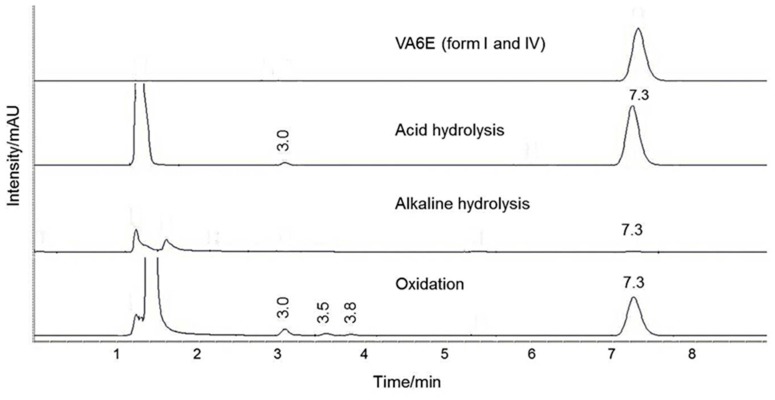
VA6E (form I and IV) chromatogram before and after 24 h application of diverse stress conditions: acid and alkaline hydrolysis and oxidation.

**Figure 10:**
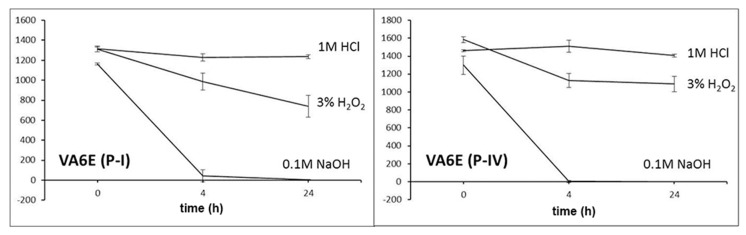
Stability of compound VA6E (polymorph I and IV) after 0, 4 and 24 h of exposition to HCl, NaOH and H_2_O_2_.

Compound VA6E (Form I and IV)—similar stability for the two polymorphs was found after application of different stress conditions (Figure 9 and Figure 10). The highest degradation occurred in the alkaline medium, as both forms degraded almost completely after 4 h of exposure to NaOH. Although the stability was higher, degradation product peaks were also detected after acid hydrolysis (t_R_ 3.0 min) and oxidation (t_R_ 3.0, 3.5 and 3.8 min).

Compound VA4J—The compound was stable after all the stress conditions studied. The VA4J chromatogram before and after 24 h application of acid and alkaline hydrolysis and oxidation showed no change in the peak area at 4.9 min t_R_ and no new peaks were detected at other retention times (data not shown).

**Figure 11:**
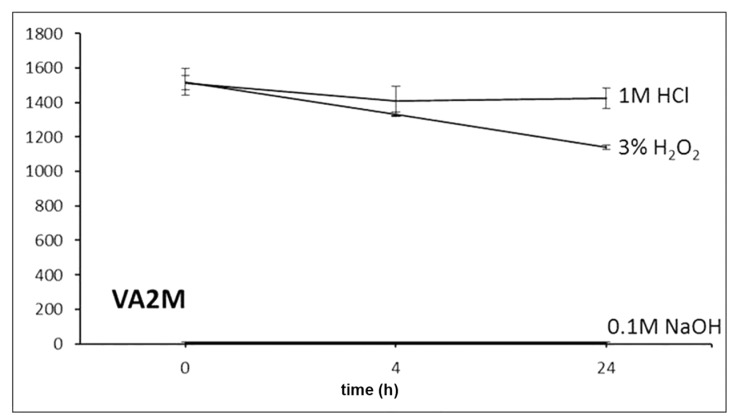
Stability of compound VA7J (polymorph I and II) after application of stress conditions.

Compound VA7J—The compound was stable to acid hydrolysis and only a minor degradation due to alkaline conditions was detected. However, it totally degraded in the presence of H_2_O_2_, as no peak was detected at its retention time at zero time of exposure to oxidation (Figure 11). A new chromatographic peak was detected at t_R_ of 1.4 min, corresponding to an oxidized form of VA7J, probably an *N*-oxide derivative.

### 2.4. Polymorphic Behaviours

To establish general polymorphic behaviours, samples of all the compounds synthetized (Figure 1) were exposed to successive cycles of melting–recrystallization using DSC. Calorimetric analyses were performed before the beginning of the degradation process (Table 6). The analysis of the thermal data revealed interesting calorimetric performance for some of these compounds. The results showed that there are five types of calorimetric behaviours:

- Behaviour I—Compounds that show a polymorphic phase transition into an amorphous or mainly amorphous solid form after a first melting–cooling scan. This behaviour was shown by several compounds: VA1C, VA2E, VA8E, series H, VA6I, VA8J, VA6M and VA8M. DSC and PXRD results of compound VA7H are shown as supplementary material (Figures S1 and S2 and Table S1).
- Behaviour II—Compounds that change into a new polymorphic form with a different *T_onset_* of fusion than the one observed at the first melting–recrystallization scan after cooling. These compounds are VA7E and VA8O. DSC and PXRD results of compound VA7E are shown as supplementary material (Figures S3 and S4, Table S2).
- Behaviour III—These compounds show a fusion process without modifications in the thermal behaviour after an initial fusion–recrystallization cycle. Consequently, there is no evidence of polymorphic behaviour. The compounds VA2C, VA2M, VA4J and VA5O belong to this group. DSC and PXRD results of compound VA2M are shown as supplementary material (Figures S5 and S6, Table S3).
- Behaviour IV—Compounds that show recrystallization or other types of phase transition of the original form into another crystalline one in the first heat scan. In the successive cycles of melting–recrystallization, the new form may not alter its crystalline form or transit to another polymorphic form. Compounds VA6E, VA7J and VA7M show this behaviour.
- Behaviour V—Compounds without fusion processes at the range of temperatures studied. The original compound shows an amorphous form that does not recrystallize into any crystalline form in the successive melting–recrystallization cycles. The compounds that showed this thermal behaviour are VA2B, VA4B, VA4C, VA1D, VA6D, series G, and VA7O.

Polymorphic behaviour is affected by reaction conditions and purification processes, as they include temperature modifications that can lead to modifications in the final solid state structure. General patterns are therefore difficult to extract as these factors vary among the 12 series. The main skeleton of the structure differs in each of these series and it has a crucial effect on the internal organization.

Some general patterns within each series of compounds can be found. All the derivatives from series B, D and G share the same behaviour (V), those compounds do not suffer transformations due to heating as they only exhibit one polymorphic form (except for compound VA8D that melts with decomposition). In the same line, compounds of series H exhibit behaviour I, which leads us to think that the conditions for the original crystalline structure to transform to the polymorph were probably not met during the synthetic process. Therefore, the structural nucleus is the main factor that determines internal organization in this series.

Interestingly, the polymorphic behaviour of some of the series changes depending on the substituent. For example, series E shows three different polymorphic behaviours depending on the attached substituent in position 5. Therefore, the substituents play an important role in the calorimetric performance of the resulting derivatives. However, no general patterns could be established in terms of the effect of substituents on internal organization, probably because of the strong effect that main nucleus modifications have among them. Thus, each series should be evaluated independently.

### 2.5. Comparative Thermal Stability

The values of thermal degradation temperatures obtained for the S and Se heteroaryl compounds are also shown in Table 6. Thermal stability is affected by intramolecular interactions and electron delocalization inside the core of the structure, as well as by interactions that might exist between the core and the substituents.

A comparison between compounds substituted with the same chemical group is hard to establish since only compounds **6**, **7** and **8** are present in most of the mentioned series. Moreover, the variability of the chemical nature of the heterocycles used enables us to observe only general trends in the analysis. For example, quinolone-based structures (series H) are less stable with *T_onset_* values of 139.1 °C, 145.8 °C and 190.2 °C for the corresponding derivatives **6**, **7** and **8**, respectively. On the other hand, the structures of series I are among the most stable with *T_onset_* over 220 °C in all three cases and ranking among the two most stable compounds in derivatives **6**, **7** and **8**.

Patterns relating to the substituent nature and thermal stability of the compounds can be spotted when focusing on those compounds with a common core structure (Series B, C, D, E, F, G, H, I, J, M, O and P).

Stability is enhanced in structures with the ability to establish hydrogen intermolecular interactions. Overall, SH (compound **2**) or SeH (compound **1**) groups improve stability dramatically. If we look at other substituents that can establish hydrogen interactions such as –Se–C(NH_2_)=NH or –S–C(NH_2_)=NH, we can also observe an increase in the thermal stability. Although compounds of series 7 and 8 are homologs except for the presence of Se or S atoms, S derivatives are more stable than Se-substituted compounds in general. 

Considering the other substituents, compounds with a nitrile (CN; compounds **5** and **6**) or methyl (CH_3_; compounds **4**) group showed lower *T_onset_* values of degradation, with this effect being more pronounced for the methyl group. Methyl-substituted structures might be less stable because they do not have the ability to establish either hydrogen interaction.

## 3. Materials and Methods

### 3.1. Chemistry

Proton (^1^H) and carbon (^13^C) NMR spectra were recorded on a Brüker 400 Ultra-shield™ spectrometer (Brüker, Rheinstetten, Germany) using DMSO-*d*_6_ or CDCl_3_ as solvent. IR spectra were recorded on a Thermo Nicolet FT-IR Nexus spectrophotometer (Thermo Nicolet, Madison, WI, USA) using KBr pellets for solid samples or NaCl plates for oil compounds. Elemental analysis was performed on a LECO CHN-900 Elemental Analyzer (LECO, Saint Joseph, MI, USA). The purity of all final compounds was 99% or higher. Chemicals were purchased from E. Merck (Darmstadt, Germany), Panreac Química S.A. (Barcelona, Spain), Sigma-Aldrich Quimica, S.A. (Madrid, Spain) and Acros Organics (Janssen Pharmaceuticalaan, Geel, Belgium). The synthesis of 34 out of the 40 compounds analysed in this work has been previously described [28,29].

#### 3.1.1. General Procedure for the Synthesis of Compounds VA6G, VA6H, VA6I and VA6J

**Scheme 1:**
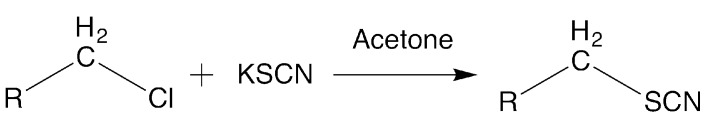
Reaction scheme to yield compounds VA6G, VA6H, VA6I and VA6J.

The synthesis (Scheme 1) was carried out from the corresponding alkyl-halide reagent and potassium-thiocyanate in acetone at reflux, as previously described. The corresponding halide reagent (1 mmol) was added to a mixture of 1.2 mmol of potassium thiocyanate in dry acetone (20 mL). The mixture was stirred at reflux for 3.5–4 h and the product was isolated by filtration after the addition of 50 mL of water and purified by washing or recrystallization.

#### 3.1.2. 3-Mercaptoquinoxalin-2-yl Carbamimidothioate Hydrochloride (VA2J)

**Scheme 2:**
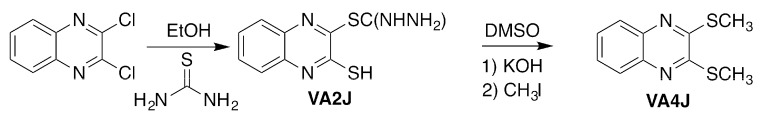
Synthesis schematics for compounds VA2J and VA4J.

To obtain compound VA2J, 2,3-dichloroquinoxaline was treated with thiourea in a 1:2.2 (reagent/thiourea) molar ratio (Scheme 2) at reflux for 4 h using absolute ethanol as solvent (20 mL). The product was purified by recrystallization from methanol. An orange powder was obtained. Yield: 42%; melting point (m.p.): 197–198 °C (direct combustion). ^1^H-NMR (400 MHz, DMSO-*d*_6_): δ 7.54 (t, 1H, *J* = 7.3 Hz, H6), 7.66–7.72 (m, 2H, H5 + H7), 7.90 (d, 1H, *J* = 8.1 Hz, H8), 9.76 + 9.95 (bs + bs, 4H, NH + NH_2_ + HCl), 15.13 ppm (bs, 1H, SH). ^13^C-NMR (100 MHz, DMSO-*d*_6_): δ 117.4 (C6), 127.4 (C7), 128.7 (C5), 131.9 (C8), 132.0 (C4a), 135.6 (C8a), 160.7 (C3), 165.4 (C2), 171.4 ppm (S–C–(NH)(NH_2_)). IR (KBr): $\tilde{\nu }$ 3380–3200 (s; N–H, N–H_2_), 1625 cm^−1^ (s; C=N). MS [*m*/*z* (% abundance)]: 194 (100), 161 (16), 150 (43), 134 (25). Elemental analysis calculated (%) for C_9_H_8_N_4_S_2_·HCl: C: 39.63, H: 3.33, N: 20.54; found: C: 39.92, H: 3.61, N: 20.19.

#### 3.1.3. 2,3-Bis(methylsulfanyl)quinoxaline (VA4J)

VA4J was synthesized by adding 1 mmol of VA2J to a suspension of 3.6 mmol of potassium hydroxide in DMSO (5 mL) and stirring for 1 h at room temperature (r.t.). Then, 4 mmol of methyl iodide were added and stirred for additional 4 h at r.t. (Scheme 2). The reaction medium was then poured into ice and the resultant precipitate was filtered and solved in dichloromethane (40 mL). The insoluble fraction was discarded and the solvent was removed under vacuum by rotatory evaporation. A yellow powder was obtained. Yield: 53%; mp: 118–119 °C. ^1^H-NMR (400 MHz, CDCl_3_): δ 2.75 (s, 6H, (CH_3_) × 2), 7.58–7.61 (m, 2H, H6 + H7), 7.91–7.96 ppm (m, 2H, H5 + H8). ^13^C-NMR (100 MHz, DMSO-*d*_6_): δ 13.7 ((CH_3_) × 2), 128.1 (C5 + C8), 129.4 (C6 + C7), 139.9 (C4a + C8a), 155.3 ppm (C2 + C3). IR (KBr): $\tilde{\nu }$ 2919 (m; C–H_aliph_), 1615 cm^−1^ (s; C=N). MS [*m*/*z* (% abundance)]: 222 (52; M^+•^), 207 (100), 192 (31), 175 (8), 160 (20), 102 (17). Elemental analysis calculated (%) for C_10_H_10_N_2_S_2_·1/2H_2_O: C: 51.87, H: 4.76, N: 12.10; found: C: 51.63, H: 4.56, N: 12.46.

#### 3.1.4. (9,10-Dioxo-9,10-dihydroanthracen-2-yl)methyl Thiocyanate (VA6G)

From 2-(chloromethyl)-9,10-dioxo-9,10-dihydroanthracen. Conditions: 3.5 h at reflux; recrystallized from ethanol. A yellow powder was obtained. Yield: 46%; m.p.: 174–175 °C. ^1^H-NMR (400 MHz, DMSO-*d*_6_): δ 4.58 (s, 2H, –CH_2_–), 7.93–7.96 (m, 3H, H1 + H3 + H4), 8.20–8.29 ppm (m, 4H, H5 + H6 + H7+ H8). ^13^C-NMR (100 MHz, DMSO-*d*_6_): δ 37.1 (–CH_2_–), 113.7 (–SCN), 127.6 (C5 + C8), 128.0 (C1), 128.3 (C4), 133.4 (C3), 133.8 (C4a), 134.1 (C6 + C7), 135.4 (C8a + C10a), 135.7 (C9a), 144.1 (C2), 183.0 ppm (C9 + C10). IR (KBr): $\tilde{\nu }$ 2149 (m, C≡N), 1671 cm^−1^ (s, C=O). MS [*m*/*z* (% abundance)]: 279 (5; M^+•^), 221 (100), 193 (51), 165 (27). Elemental analysis calculated (%) for C_16_H_9_NO_2_S: C: 68.80, H: 3.25, N: 5.01; found: C: 68.59, H: 3.31, N: 4.82.

#### 3.1.5. Quinolin-2-ylmethyl Thiocyanate (VA6H)

The commercially available 2-(chloromethyl)quinoline hydrochloride was treated with basic water (50 mL) in order to obtain the free base, which was used for the synthesis of VA6H. Conditions: 4 h at reflux. The precipitate was washed with ethyl ether (20 mL). A brown powder was obtained. Yield: 41%; m.p.: 80–81 °C. ^1^H-NMR (400 MHz, DMSO-*d*_6_): δ 4.74 (s, 2H, –CH_2_–), 7.63–7.66 (m, 2H, H3 + H6), 7.80 (t, 1H, *J* = 7.8 Hz, H7), 8.01 (d, 2H, *J* = 8.6 Hz, H5 + H8), 8.44 ppm (d, 1H, *J* = 8.5 Hz, H4). ^13^C NMR (100 MHz, DMSO-*d*_6_): δ 40.0 (–CH_2_–), 113.9 (–SCN), 121.8 (C3), 127.8 (C6), 127.9 (C4a), 128.8 (C5), 129.3 (C8), 131.0 (C7), 138.2 (C4), 147.8 (C8a), 156.4 ppm (C2). IR (KBr): $\tilde{\nu }$ 3058 (m; C–H_arom_), 2946 (m; C–H_aliph_), 2147 (s; C≡N), 1655 cm^−1^ (m; C=N). MS [*m*/*z* (% abundance)]: 200 (100; M^+•^), 174 (6), 142 (100), 128 (9), 115 (47). Elemental analysis calculated (%) for C_11_H_8_N_2_S·1/2H_2_O: C: 63.08, H: 4.30, N: 13.99; found: C: 62.96, H: 4.42, N: 13.15.

#### 3.1.6. (6,7-Dimethoxy-2-oxo-2*H*-chromen-4-yl)methyl Thiocyanate (VA6I)

From 4-(bromomethyl)-6,7-dimethoxy-2-oxo-2*H*-chromene. Conditions: 4 h at reflux. The product was washed with ethyl ether and dichloromethane (20 mL of each). A white powder was obtained. Yield: 70%; m.p.: 204–205 °C. ^1^H-NMR (400 MHz, DMSO-*d*_6_): δ 3.83 + 3.88 (s, 6H, (–OCH3) × 2), 4.56 (s, 2H, –CH_2_–), 6.41 (s, 1H, H3), 7.13 (s, 1H, H5), 7.38 ppm (s, 1H, H8). ^13^C-NMR (100 MHz, DMSO-*d*_6_): δ 33.6 (–CH_2_–), 57.1 + 57.3 ((–OCH_3_) × 2), 101.5 (C8), 107.2 (C5), 110.0 (–SCN), 113.3 (C3), 114.0 (C4a), 146.9 (C6), 150.4 (C8a), 150.7 (C7), 153.8 (C4), 160.8 ppm (C2). IR (KBr): $\tilde{\nu }$ 3065 (w; C–H_arom_), 2999–2849 (m; C–H_aliph_), 2154 (s; C≡N), 1711 cm^−1^ (C=O). MS [*m*/*z* (% abundance)]: 277 (100; M^+•^), 234 (6), 219 (27), 191 (69), 147 (18). Elemental analysis calculated (%) for C_13_H_11_NO_4_S: C: 56.31, H: 4.00, N: 5.05; found: C: 56.40, H: 3.98, N: 4.88.

#### 3.1.7. Quinoxalin-2,3-diyldimethanediyl Bisthiocyanate (VA6J)

From 2,3-bis(bromomethyl)quinoxaline. Conditions: 4 h. at reflux. The product was washed with ethyl ether (20 mL). A brown powder was obtained. Yield: 88%; m.p.: 158–159 °C (direct combustion). ^1^H-NMR (400 MHz, DMSO-*d*_6_): δ 4.98 (s, 4H, (–CH_2_–) × 2), 7.91–7.93 (m, 2H, H6 + H7), 8.11–8.14 ppm (m, 2H, H5 + H8). ^13^C-NMR (100 MHz, DMSO-*d*_6_): δ 37.7 ((–CH_2_–) × 2), 112.0 ((–SCN) × 2), 128.4 (C5 + C8), 128.4 (C6 + C7), 143.7 (C4a + C8a), 160.3 ppm (C2 + C3). IR (KBr): $\tilde{\nu }$ 2926 (m; C–H_aliph_), 2155 (s; C≡N), 1563–1485 cm^−1^ (m; C–C_arom_). MS [*m*/*z* (% abundance)]: 272 (36; M^+•^), 245 (14), 214 (100), 187 (15), 170 (13), 155 (30), 129(13). Elemental analysis calculated (%) for C_12_H_8_N_4_S_2_: C: 52.92, H: 2.96, N: 20.57; found: C: 52.7, H: 3.07, N: 20.77.

### 3.2. Biological Evaluation

The antitumoural evaluation of 34 out of the 40 compounds analysed in this work has been previously described [28,29].

#### 3.2.1. Cell Culture

Cell lines were purchased from the American Type Culture Collection (ATCC, Barcelona, Spain). MCF7 cell lines were grown in Roswell Park Memorial Institute (RPMI) medium (Gibco, Madrid, Spain) supplemented with 10% fetal bovine serum (FBS; Gibco), 100 units/mL penicillin and 100 mg/mL streptomycin (Gibco, Madrid, Spain). 184B5 cells were grown in DMEM/F12 medium supplemented with 5% FBS, 1x ITS (Lonza, Barcelona, Spain), 100 nM hydrocortisone (Sigma Aldrich, Madrid, Spain), 2 mM sodium pyruvate (Lonza), 20 ng/mL EGF (Sigma Aldrich, Madrid, Spain), 0.3 nM trans-retinoic acid (Sigma-Aldrich) 100 units/mL penicillin and 100 mg/mL streptomycin. Cells were maintained at 37 °C and 5% CO_2_.

#### 3.2.2. Cell Viability

Compounds were dissolved in DMSO at a concentration of 0.01 M. Serial dilutions were prepared with non-supplemented medium. The cytotoxic effect of each compound was tested at eight different concentrations ranging between 0.01 and 100 µM.

A total of 10^4^ cells/well in 96 well-plates were treated with either DMSO or increasing concentrations of the corresponding compound for 72 h and cell viability was determined using the MTT (3-(4,5-dimethylthiazol-2-yl)-2,5-diphenyltetrazolium bromide) (Sigma Aldrich, Madrid, Spain) assay [33]. Briefly, cells were incubated with 50 µL of MTT (2 mg/mL stock) for 4 h. The medium was then removed by aspiration and the formazan crystals were dissolved in 150 µL of DMSO. The absorbance was measured at 550 nm in a microplate reader (Sunrise, Tecan, Männedorf, Switzerland). Results are expressed as GI_50_, the concentration that reduces by 50% the growth of treated cells with respect to untreated controls, TGI, the concentration that completely inhibits cell growth, and LC_50_, the concentration that kills 50% of the cells. Data were obtained from at least three independent experiments performed in quadruplicate.

### 3.3. Thermal Analysis

The thermogravimetric (TG) studies were carried out with a Perkin–Elmer TGA-7 (Perkin Elmer, Inc. Watham, MA, USA) which was calibrated with alumel and nickel at 10 °C·min^−1^. The calibration of the oven temperatures was carried out automatically and mass calibrated with a certified sample of 10 mg (ASTM E617, provided by Perkin Elmer, Inc. Watham, MA, USA). TG analyses were performed under a nitrogen atmosphere with a gas flow of 40 mL·min^−1^, from 50 to 350 °C, at a heating rate of 10 °C·min^−1^, using a sample of approximately 3 mg, in order to obtain the values of the *T_onset_* and T_max_ of the degradation process as well as any associated mass loss.

For calorimetric studies a Perkin–Elmer DSC diamond calibrated with indium and zinc (provided by Perkin–Elmer (Perkin Elmer, Inc. Watham, MA, USA) and fabricated according to guideline ISO35) at 10 °C·min^−1^ and nitrogen flow of 20 mL·min^−1^ was employed. The gas connected to the equipment was nitrogen with a purity of 99.999%. Calorimetric analyses were carried out in aluminium capsules for volatilities of 10 μL, at a heating rate of 10 °C·min^−1^, using a sample of approximately 3 mg, in order to establish the *T_onset_*, T_max_ and the enthalpy of fusion ∆H_f_.

The calorimetric analyses started with the study of the thermal behaviour of the compounds before the process of degradation in order to evidence the possible existence of polymorphism. For this preliminary study, samples of all the compounds were exposed to successive cycles of heating–cooling. All samples were heated from 50 °C to temperatures 15–20 °C below *T_onset_* of the degradation process, in order to ensure that compounds were not degraded (Table 6). After melting the samples, they were left at room temperature for enough time to allow the compounds to recrystallize before the successive thermal process. Additional cycles of heating–cooling were performed if polymorphism was detected.

### 3.4. Powder X-ray Diffraction

The X-ray diffraction experiments were performed by using a Bruker D8 Advance diffractometer, under the conditions of a step size of 0.02° and 1 s time/step, from 5° < 2θ < 40°. The equipment consisted of an X-ray generator with a Cu anode (radiation Cu Kα = 1.54 Å, 40 kV, 30 mA) and a scillintator detector. Experiments were performed in reflectance mode. Measurements were carried out on samples laid on a glass slide without additional manipulation. The preferential grain orientations that are likely to exist in these compounds could not be avoided due to the absence of grinding of the samples and also to the impossibility of using spinning capillary in our equipment.

### 3.5. Chromatography

Chromatographic studies were performed using an HPLC/UV-DAD (HP 1200, Agilent Technologies. Santa Clara, CA, USA) with a C18 column (Gemini-NX 150 × 4.6 mm, 5 µm) from Phenomenex (Phenomenex. Torrance, CA, USA). The mobile phase was a mixture of acetonitrile/water (80:20; *v*/*v*) for compound VA4J, (50:50; *v*/*v*) for VA6E and (80:20, 0.1% formic acid; *v*/*v*) for compound VA7J. The injection volume was 50 µL and the flow rate was 1 mL/min. Chromatography was performed at 35 °C. UV-DAD detection was established at λ = 254 nm.

In the assessment of linearity, calibration curves were plotted in the range of 3–119 µg/mL for VA6E and 0.8–59.5 µg/mL for VA4J and VA7J. Calibration curves were evaluated by the analysis of the distribution properties of the residuals, correlation coefficient *r*^2^ > 0.990, slope of the linear calibration curve statistically different from 0 (*p* = 95%) and the intercept not statistically different from 0 (*p* = 95%). Precision (as RSD%) of the method was evaluated by analysing three replicate calibration samples at five concentration values (including lower, medium and higher value) of the calibration curve.

Stock standard solutions containing 1 mg/mL of compound VA4J, VA6E and VA7J were prepared by diluting each compound in a mixture of acetonitrile/water (50:50; *v*/*v*). Calibration samples in the mobile phase were prepared by the dilution of the stock standard solutions.

For stability evaluation in stress conditions, working solutions of 47.6 µg/mL were prepared by diluting 20 µL of each standard solution in 200 µL of acetonitrile and 200 µL of (a) 1 M HCl, (b) 0.1 M NaOH and (c) 3% H_2_O_2_. Data were recorded at zero time and after 4 and 24 h of exposure.

## 4. Conclusions

The thermal characterization of compounds with antitumour potency is crucial to further continue the drug discovery process. In order to predict the non-desired transformations that might occur in the pharmaceutical manufacturing process, the polymorphism of the most promising compounds has been studied.

Regarding the results of differential scanning calorimetry, powder X-ray diffraction and the stability studies under stress conditions:

- Compound VA6E: temperature conditions have to be carefully selected as solid state behaviour includes four different polymorphs. In terms of stability, alkaline, acid and oxidation media are not suggested as they cause degradation.
- Compound VA7J: oxidation conditions and desolvation have to be taken into account as they lead to immediate degradation and crystal structure modification, respectively.
- Compound VA4J: there is no evidence of polymorphism and stress conditions do not affect the solid-state structure.

Taken together we can conclude that compounds VA6E and VA7J present some obstacles to further development as drugs as both temperature and stress conditions affect the original structure. On the other hand, compound VA4J proved to be safe to work with under the conditions studied. Therefore, this compound is of special interest for further development as it shows promising activity, selectivity and stability.

In addition, it has been found that the polymorphic behaviour as well as the thermal stability of these compounds can be modified by the introduction of different functional groups in the heterocycle under consideration. According to our data, the incorporation of a selenol or thiol group improves the thermal stability.

## Figures and Tables

**Table 1:** Average values of GI_50_, TGI and LD_50_ (μM) and calculated SI.

Compound	Cell Lines						SI
	MCF-7			184B5			
	GI_50_	TGI	LD_50_	GI_50_	TGI	LD_50_	
VA2J	2.20	13.90	44.02	12.45	15.97	20.48	5.66
VA4J	1.02	>100	>100	52.45	>100	>100	51.25
VA6G	3.59	23.95	67.27	14.29	34.32	64.84	3.99
VA6H	8.53	14.83	24.18	27.76	75.84	>100	3.25
VA6I	4.51	>100	>100	2.26	>100	>100	0.50
VA6J	18.50	50.55	83.93	39.77	69.80	94.62	2.15
MSA	1.28	3.43	>100	1.79	3.84	>100	1.40

**Table 2:** Fusion–recrystallization of compound VA6E.

Scan	Form I		Recrystallization		New Polymorphs	
	*T_onset_*(°C)	∆*H* (J g^−1^)	*T_onset_* (°C)	∆*H* (J g^−1^)	*T_onset_* (°C)	∆*H* (J g^−1^)
1st	97.2	7.9	98.5	−8.8	101.9	75.6
	(Form I)				(Form II)	
2nd	-	-	-	-	100.7	80.3
					(Form III)	
3rd	-	-	-	-	100.1	76.7
					(Form IV)	
4th	-	-	-	-	99.9	76.0
					(Form IV)	

**Table 3:** Fusion–recrystallization of compound VA4J.

	*T_onset_* (°C)	∆*H* (J g^−1^)	
1st scan	119.84	93.58	Form I
2nd scan	119.59	93.91	Form I

**Table 4:** Fusion–recrystallization of compound VA7J.

Behaviour III	*T_onset_* (°C)	∆*H* (J g^−1^)	
1st scan	173.8	−88.4	Form I
	218.2	18.7	Form II
2nd scan	219.7	17.4	Form II

**Table 5:** Thermal data obtained by DSC and TG.

Ref.	R	DSC				TG
		Scan	T_o_/°C	∆*H_f_*/J g^−1^	Behaviour	T_o_/°C
**Series B**						
VA2B	SH	1st	-	-	V	263.3
VA4B	SCH_3_	1st	-	-	V	103.5
**Series C**						
VA1C	SeH	1st	175.5	91.3	I	218.4
		2nd	-	-		
VA2C	SH	1st	178.8	85.9	III	224.7
		2nd	178.6	86.6		
VA4C	SCH_3_	1st	-	-	V	131.8
**Series D**						
VA1D	SeH	1st	-	-	V	244.7
VA6D	SCN	1st	-	-	V	205.0
VA8D	SC(NHNH_2_)	1st	196.7	−90.3	*	193.1
**Series E**						
VA2E	SH	1st	148.8	34.0	I	266.2
		2nd	-	-		
VA6E	SCN		97.2	7.8	IV	184.1
		1st	98.4	−8.8		
			101.9	75.6		
		2nd	100.7	80.3		
		3th	100.1	76.7		
		4th	99.9	76.0		
VA7E	SeC(NHNH_2_)	1st	183.8	21.5	II	205.2
		2nd	177.2	28.1		
		3th	-	-		
VA8E	SC(NHNH_2_)	1st	196.1	33.8	I	215.1
			208.5	−37.4		
		2nd	-	-		
**Series F**						
VA7F	SeC(NHNH_2_)	1st	214.4	−63.6	*	214.7
VA8F	SC(NHNH_2_)	1st	198.3	−64.6	*	190.8
**Series G**						
VA6G	SCN	1st	-	-	V	238.3
VA7G	SeC(NHNH_2_)	1st	-	-	V	198.2
VA8G	SC(NHNH_2_)	1st	-	-	V	243.7
**Series H**						
VA4H	SCH_3_	1st	54.9	75.7	I	105.6
		2nd	-	-		
VA6H	SCN	1st	78.9	116.6	I	139.1
		2nd	-	-		
VA7H	SeC(NHNH_2_)	1st	113.7	42.0	I	145.8
		2nd	-	-		
VA8H	SC(NHNH_2_)	1st	176.2	49.3	I	190.2
		2nd	-	-		
**Series I**						
VA6I	SCN	1st	234.9	110.8	I	246.9
		2nd	-	-		
VA7I	SeC(NHNH_2_)	1st	230.1	30.1	*	226.4
			234.4	−14.4		
VA8I	SC(NHNH_2_)	1st	263.2	39.6	*	272.4
			265.4	−36.7		
**Series J**						
VA2J	SH/SC(NHNH_2_)	1st	206.5	−63.9	*	199.2
VA4J	SCH_3_		119.84	93.58	I	197.42
		1st	119.59	93.91		
VA6J	SCN	2nd	167.6	−227.0	*	165.0
VA7J	SeC(NHNH_2_)	1st	173.8	−88.4	IV	From 228
			218.2	18.5		
		2nd	219.7	17.4		
		3th	219.6	17.4		
VA8J	SC(NHNH_2_)	1st	222.9	31.3	I	251.05
			224.1	−95.3		
		2nd	-	-		
**Series M**						
VA2M	SH	1st	113.6	68.1	III	166.0
		2nd	110.2	70.7		
VA6M	SCN	1st	143.3	50.8	I	164.8
		2nd	-	-		
VA7M	SeC(NHNH_2_)	1st	211.6	34.4	IV	255.8
			216.0	−68.7		
			220.0	4.4		
		2nd	219.7	10.3		
		3th	219.8	14.8		
VA8M	SC(NHNH_2_)	1st	228.7	116.7	I	233.1
		2nd	-	-		
**Series O**						
VA5O	SeCN	1st	67.7	76.0	III	141.7
		2nd	65.6	66.2		
VA7O	SeC(NHNH_2_)	1st	-	-	V	185.9
VA8O	SC(NHNH_2_)	1st	194.7	21.1	II	201.8
		2nd	199.8	45.8		
		3rd	-	-		
**Series P**						
VA5P	SeCN	1st	161.3	79.6	*	149.4
VA7P	SeC(NHNH_2_)	1st	180.2	−84.7	*	175.8
VA8P	SC(NHNH_2_)	1st	200.6	1.7	*	199.8
		2nd	210.1	−60.4		

* fusion with decomposition.

**Table 6:** Limit of Quantification (LOQ), linearity data and within-day precision.

	t_R_ (min)	LOQ (μg/mL)	Range (μg/mL)	Curve Equation	*r*^2^	Slope Interval (*p* = 95%)	Within-Day Precision	
							[μg/mL]	%RSD (*n* = 3)
VA4J	4.9	0.8	0.8–59.5	*y* = 97.7*x* − 51.1	0.999	96.0–99.9	0.8	5.6
							1.0	5.5
							6.0	4.6
							23.8	2.6
							59.5	0.5
VA6E	7.3	3.0	3.0–119.0	*y* = 26.2*x* − 59.2	0.996	25.3–27.1	3.0	0.8
							6.0	2.4
							11.9	3.9
							47.6	1.5
							119.0	0.7
VA7J	1.1	0.8	0.8–59.5	*y* = 97.5*x* + 37.1	0.999	94.9–100.0	0.8	6.5
							1.0	3.9
							6.0	10.5
							23.8	1.7
							59.5	0.9

## Supplementary Materials

supplementary material are available online.
