# Supplementary material for: Antitumoural Sulphur and Selenium Heteroaryl Compounds: Thermal Characterization and Stability Evaluation
Source: Molecules. 2017 Aug 8;22(8):1314. doi: 10.3390/molecules22081314 (PMC6152329; doi:10.3390/molecules22081314)
Supplement: Supplementary file 1 [file molecules-22-01314-s001.pdf]

molecules

Appendix A: supplementary data

Compound VA7H (behaviour I)

Differential scanning calorimetry. As an example, the DSC results of compound VA7H are shown in Figure S1 and Table S1. During the first thermal scan (curve A), two process were observed. The first one is typical of a glass transition (Tg) and then, an endothermic melting process happened, demonstrating that amorphous and crystalline forms coexist in the original compound. After cooling the sample, through a polymorphic phase transition, the initial form (Form I) changed into an amorphous one which did not show more fusion processes (Form II, curve B). The complete thermogram shows that other processes at another temperature (higher or lower) can be excluded.

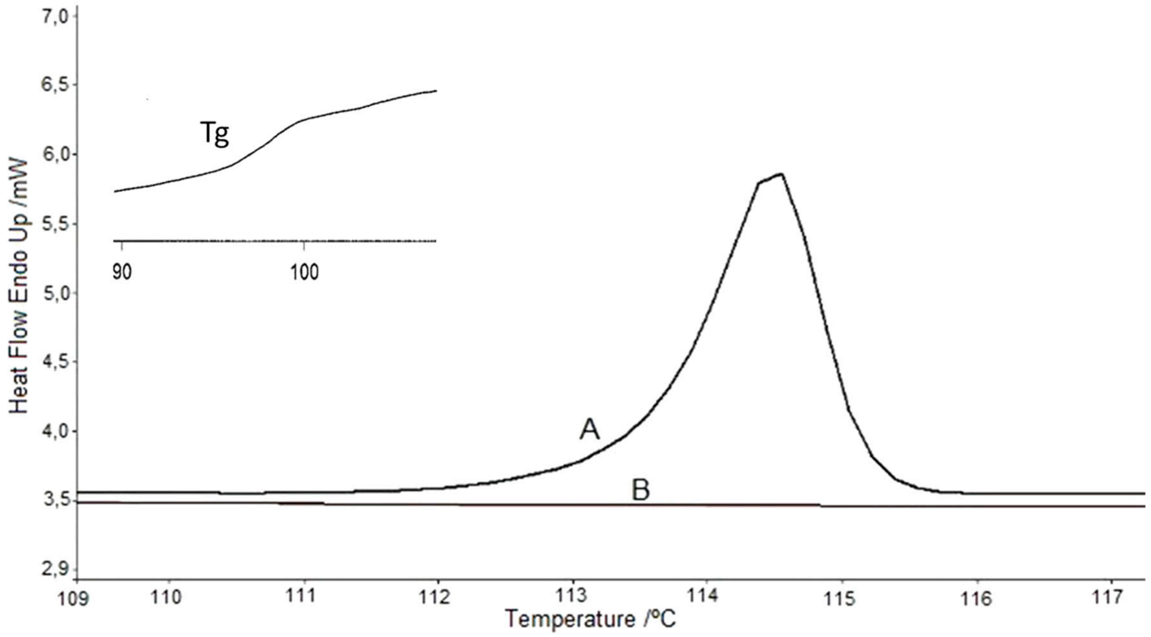

Figure S1. DSC of compound VA7H (curve A: first scan; curve B: second scan)

Table S1. Fusion-recrystallization of compound VA7H

Behaviour I	T_{onset} (°C)	Δ*H* (Jg^{-1})	
1st scan	113.7	42.1	Form I
2nd scan	-	-	Form II

Regarding *XRD results*, two different polymorphic forms were detected: Form I with a low degree of crystallinity and Form II, mostly amorphous form. Peaks at 2θ=5.9, 21.3, 22.4, 23.5, 26.9, 27.9, 29.7 and 30.0 in diffractograms differed from one another as shown in Figure S2.

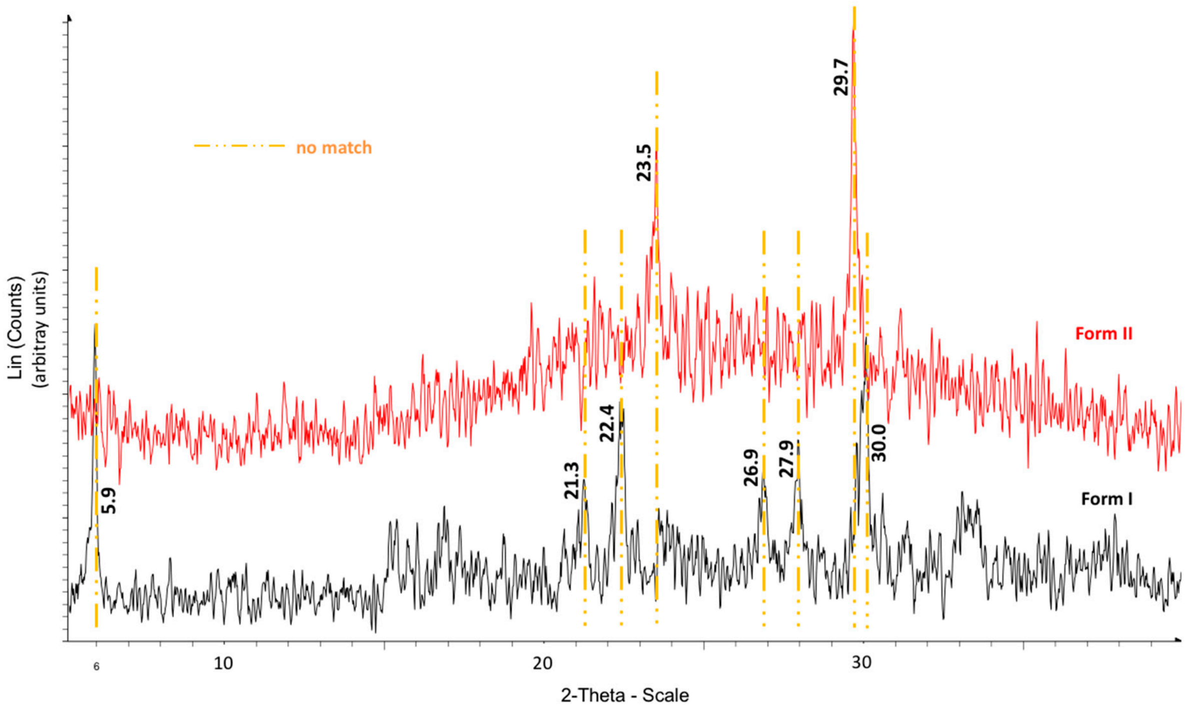

Figure S2. X-ray diffractogram for VA7H (Form I: original polymorph and form II: mostly amorphous form)

Compound VA7E (behaviour II)

After a first thermal curve of compound VA7E (curve A in Figure S3) with a typical endothermic process of Form I (Table S2), in a second scan, a new endothermic process happened at a lower temperature (curve B), with the appearance of a new polymorph (Form II) that turned into an amorphous form after cooling. No eutectic peak at lower temperatures was detected.

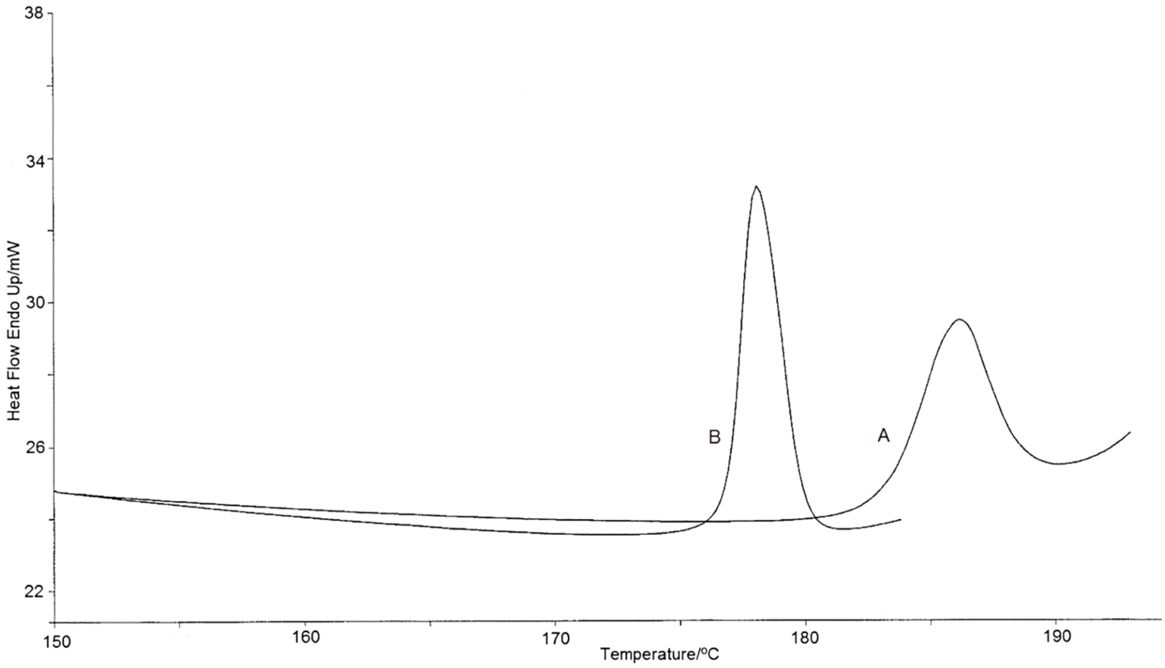

Figure S3. DSC of compound VA7E (curve A: first scan; curve B: second scan)

Table S2. Fusion-recrystallization of compound VA7E

Behaviour II	T_{onset} (°C)	ΔH (Jg^{-1})	
1st scan	183.8	21.5	Form I
2nd scan	177.2	28.1	Form II
3rd scan	-	-	Amorphous form

Two polymorphic forms were detected in the *X-ray diffraction studies* (Figure S4). Peaks at 2θ=20.0, 27.3, 32.3 and 32.6 only appeared in Form II whereas signal at 2θ=22.1 was exclusive of Form I.

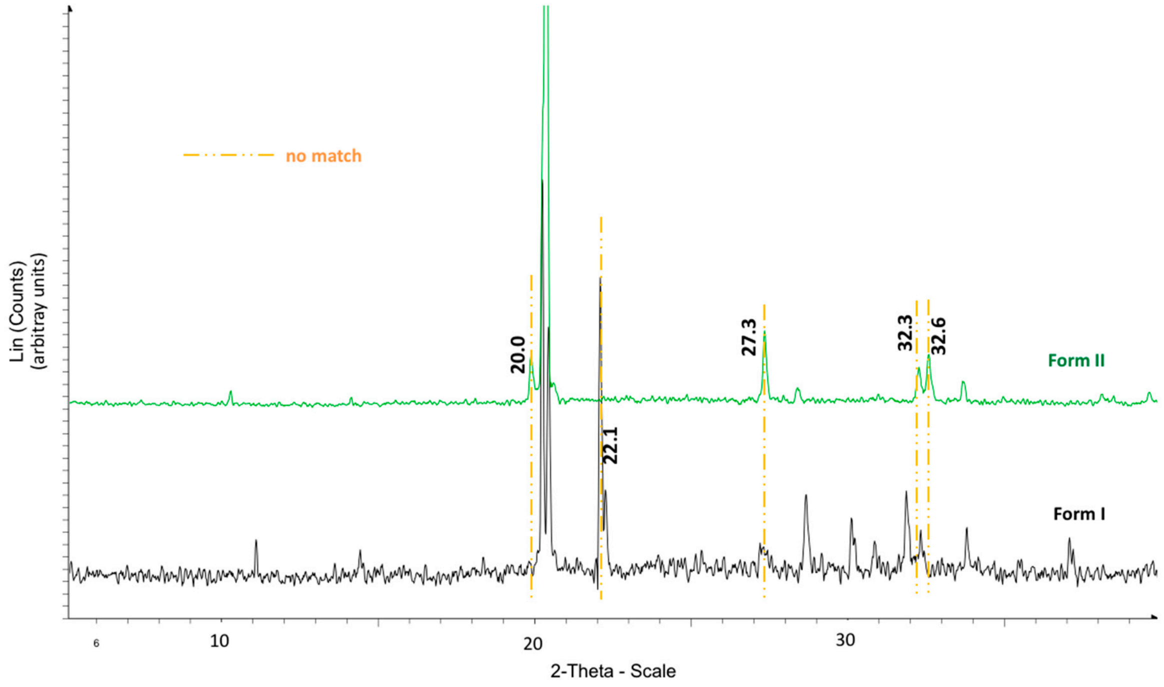

Figure S4. X-ray diffractogram for compound VA7E. Form I shows the diffractogram of the original polymorph and II the one obtained after heat treatment

Compound VA2M (behaviour III):

This compound shows a fusion process without modifications in the thermal behaviour after successive fusion-recrystallization cycles (Figure S5 and Table S3). Consequently, there is no evidence of polymorphic behaviour.

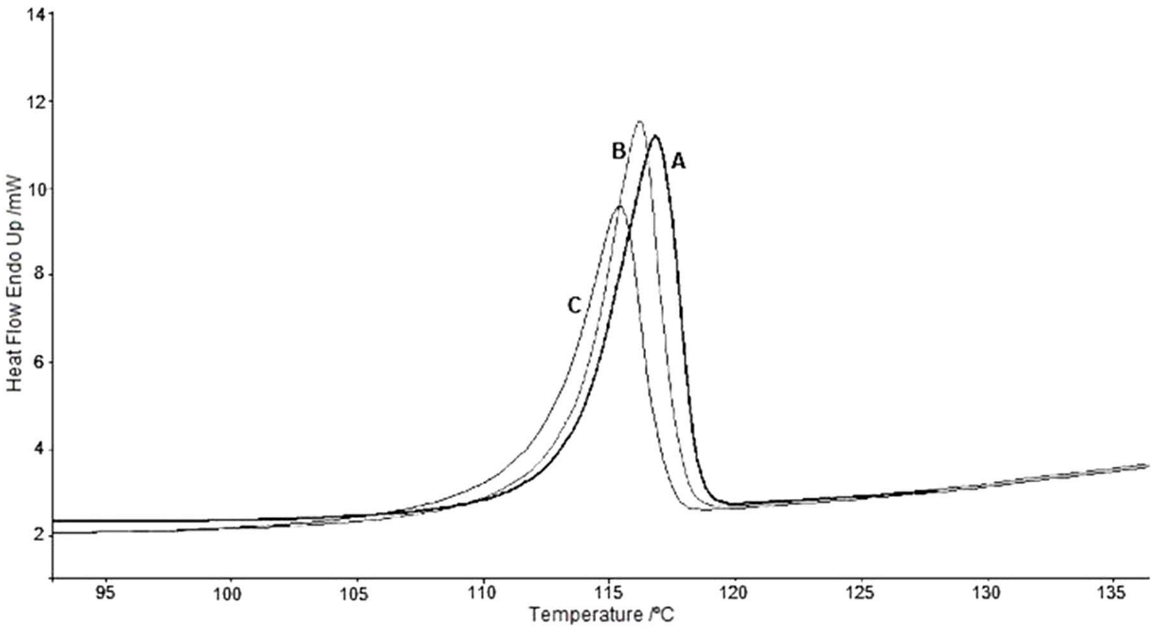

Figure S5. DSC of compound VA2M. Curve A: first scan; curve B: second scan; curve C: third scan

Table S3. Fusion-recrystallization of compound VA2M

Behaviour III	T_{onset} (°C)	Δ*H* (Jg^{-1})	
1st scan	113.7	68.1	Form I
2nd scan	110.2	70.7	Form I

X-ray powder diffractometry: to further confirm the absence of polymorphism, both samples were analysed by X-ray powder diffractometry. As it can be observed in Figure S6, no differences were found in the positions of the peaks between the original sample (after the synthesis and purification) and the one obtained after melting and a subsequent recrystallization process. The differences in the intensities of some of the peaks were probably due to some preferred orientation in the samples derived from the absence of random orientation of crystal grains in space. Anyway, we could observe a very good match between peak positions in both diffractograms.

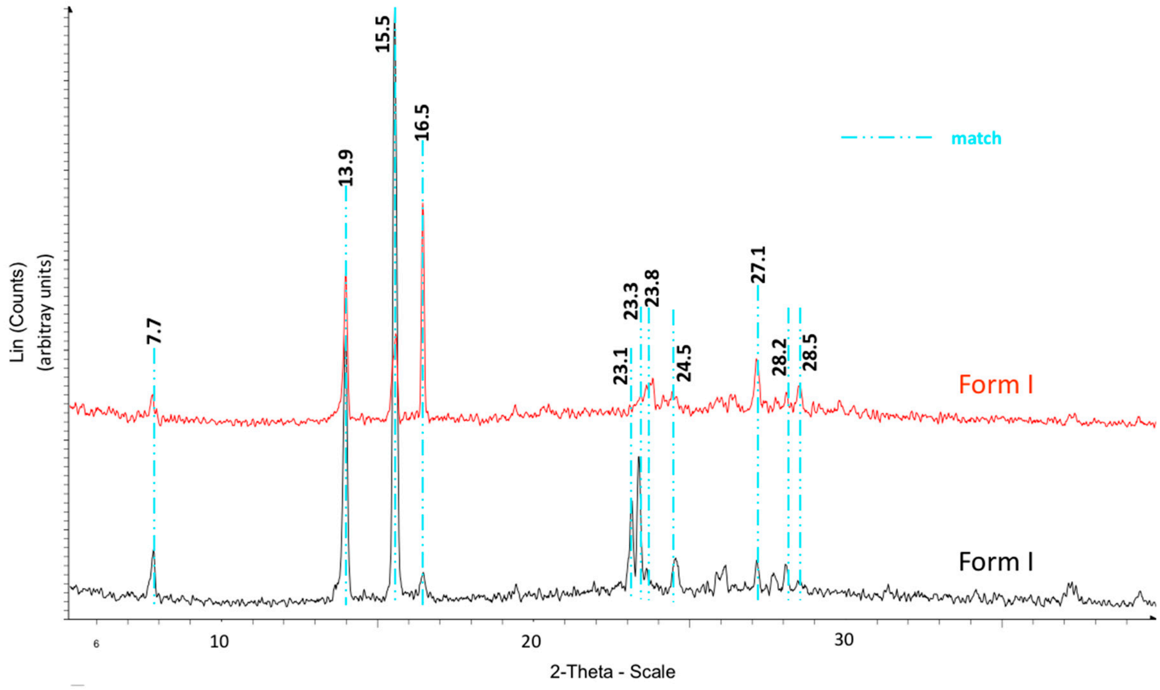

Figure S6. X-ray diffractogram for compound VA2M diffractogram of the original compound before (black XRD) and after heat treatment (red XRD)
